# Positive evidence for clinical pharmacist interventions during interdisciplinary rounding at a psychiatric hospital

**DOI:** 10.1038/s41598-021-92909-2

**Published:** 2021-07-01

**Authors:** Matej Stuhec, Valentina Tement

**Affiliations:** 1 Department of Clinical Pharmacy, Ormoz Psychiatric Hospital, Ptujska cesta 33, 2270 Ormoz, Slovenia; 2 University of Ljubljana, Faculty of Pharmacy, Askerceva cesta 7, 1000 Ljubljana, Slovenia; 3 Department of Pharmacology, Faculty of Medicine Maribor, University of Maribor, Maribor, Slovenia

**Keywords:** Health care, Medical research

## Abstract

Clinical pharmacists have not yet become an integral part of interdisciplinary ward rounds in most psychiatric hospitals across the European Union. This retrospective observational pre-post study examined the impact of clinical pharmacist recommendations in an interdisciplinary medical team during psychiatric hospital rounding. The study included all patients in a Slovenian psychiatric hospital who were hospitalized 2019–2020. The clinical pharmacist made 315 recommendations for a total of 224 participants (average age *M* = 59.4, median = 56). Psychiatrists accepted 295 (93.7%) of the recommendations. After the recommendations, the number of expressed and potential drug-related problems decreased in 166 (93.8%) and 129 (93.8%) interventions, respectively. Three months after discharge, 222 accepted recommendations were continued (70.5%). The most common recommendations were related to antipsychotics (19.4%, N = 61) followed by antidepressants (16.8%, N = 53). Including a clinical pharmacist in the interdisciplinary ward rounds at a psychiatric hospital reduced the number of expressed and potential drug-related problems with a very high recommendation acceptance rate. These results are the first in Central Europe to explore the benefits of including a clinical pharmacist in ward rounding.

## Introduction

Mental disorders pose a high disease burden and health costs, which continue to increase with significant health, social, ethical and economic consequences in all countries. Mental disorders account for 13% of the global disease burden, and major depression alone is expected to be the largest contributor by 2030^1^. Wittchen et al. report that the most frequent disorders in the European Union are anxiety disorders (14.0%), insomnia (7.0%), major depression (6.9%), somatoform disorders (6.3%), alcohol and drug dependence (> 4%), attention-deficit hyperactivity disorder among youth (5%), and dementia (1–⁠30%, depending on age)^2^. Although there are non-pharmacological treatment options (e.g., psychotherapy), pharmacotherapy is often necessary. Patients with mental disorders are often treated with several medications (i.e., polypharmacy) and for several diseases. This opens the risk of unnecessary polypharmacy, potentially inappropriate medications (PIMs) and irrational medication combinations. In spite of lacking evidence, patients are often treated with psychotropics to manage several conditions (e.g., insomnia, depression, and behavioral symptoms of dementia treatment)^3,4^. Consequently, there is a need for well-designed studies with strong ecological validity in clinical practice to minimize medication-related problems and plan appropriate strategies.

Inpatients in psychiatric hospitals often have treatment-resistant conditions, are concurrently treated for other diseases (e.g., diabetes, infectious diseases, seizures), and receive medication combinations that are not always evidence-based. This creates a risk of inappropriate polypharmacy, which can result in inappropriate treatment, adverse events, and treatment failures^3^. Psychotropics have a particularly high risk of potential drug-drug interactions (pDDIs), adverse events (e.g., weight gain) and PIM prescribing. A retrospective descriptive chart study in a Slovene psychiatric hospital found that 47% and 22% of the prescriptions were concomitant prescriptions of two and three antipsychotics, respectively. It also found many inappropriate combinations, including antipsychotic polypharmacy (APP)^4^. Clozapine prescriptions in psychiatric hospitals are less frequent than expected in patients with two unsuccessful treatments and many patients are treated with APP before clozapine initiation^5^.

A promising approach to address these drawbacks for patients with mental health disorders is collaborative care that involves a clinical pharmacist, which first became more widespread in the 1990s in the US, where it demonstrated improved outcomes and medication adherence in primary care settings^6,7^. Outside the US, Stuhec et al. report a decrease in medication-related problems (number of PIMs and pDDIs) and an improvement in the patients’ quality of life following a clinical pharmacist’s medication review service in a Slovenian nursing home^8^. There is limited research on whether these benefits also apply to psychiatric hospitals, in which patients often require complex treatments, and most studies on the topic have been conducted in the US^6–9^. A retrospective analysis in a Californian acute care psychiatric hospital reported a positive impact of clinical pharmacist interventions during ward rounds. The interventions had a 92.5% acceptance rate. Most frequently, they were medication discontinuations (38.5%), laboratory monitoring (26%), and medication order modification (13.5%). The 200 clinical pharmacist interventions were also associated with significant cost savings and cost avoidance^9^. A 2002 study of US hospitals (*N* = 1081) found a significant inverse relationship between medical errors and pharmacist participation on medical rounds (slope = −0.6974303, *p* < 0.001), as well as with pharmacist-provided admission histories (slope = −1.6021493, *p* < 0.001)^10^. While not obtained from a randomized study, these results suggest that pharmacist participation in ward rounds may be an important way to optimize pharmacotherapy in psychiatric hospitals. A 2020 systematic review (N = 64) found that the incorporation of psychiatric pharmacist input into interprofessional healthcare teams was the most common pharmacist practice in psychiatric and neurological settings and was associated with significant improvements in patient-level outcomes. However, none of the included papers from the European Union specifically evaluated clinical pharmacist inclusion in daily interdisciplinary ward rounding^11^.

Because clinical pharmacists are usually not part of interdisciplinary medical teams in psychiatric settings in the Central Europe, this paper sets out to examine the impact of clinical pharmacist interventions during hospital rounds in a Slovenian psychiatric hospital.

## Methods

### Study setting

This study was conducted in the Ormoz Psychiatric Hospital (*Psihiatrična bolnišnica Ormož*), which covers approximately 200,000 inhabitants in Northeastern Slovenia. It has 120 hospital beds and provides treatment for all mental disorders, a community psychiatric service, and a daily hospital and outpatient service. It receives approximately 1000 inpatients annually with an average length of stay of 28 days. It has six wards: a psychogeriatric ward, a daily hospital center, an addiction treatment center, two closed wards, and an open ward for prolonged treatment. Daily hospital activities include interdisciplinary team activities (e.g., ward rounding) and individual activities (e.g., daily conversation between patients and various healthcare professionals, occupational therapy activities, psychotherapy).

The interventions described in this study were provided by a single clinical pharmacist, who has been board-certified since 2014 and has been working in the hospital for over six years as a ward clinical pharmacist. The daily work includes ward rounds, medication reviews, medication error reports, and educating patients and caregivers. The pharmacist participates in six different ward rounds at each of the six hospital departments weekly, spending approximately an hour in ward rounding daily. During the study period, ward rounding was done 1–2 times per week for each ward and included all interdisciplinary team members: a psychiatrist, nurse, clinical pharmacist, psychologist, occupational therapist, and a social worker. Other ward staff were occasionally also included, as well as residents (e.g., psychiatrists, psychologists and clinical pharmacists) and students (e.g., nursing, pharmacy, psychology and occupational therapy). Ward roundings covered 10–20 patients in 60–90 min, including a discussion and potential decisions within the interdisciplinary team, such as changes in patient pharmacotherapy. The hospital employed one board-certified clinical pharmacist specialist. Clinical pharmacists may suggest interventions to ward psychiatrists during the roundings. Each intervention is recorded in electronic patient records. Clinical pharmacists monitor all interventions and adjust them at subsequent ward roundings if necessary. They play an important role in the team by contributing expertise through medication recommendations and by designing and monitoring treatment plans. They do not measure clinical outcomes, but do have access to patient health data. The inclusion of a clinical pharmacist in the interdisciplinary team is a care standard since 2018 (as per the Slovenian Pharmacy Act), but not practiced in all psychiatric hospitals. The clinical pharmacist in our study was required to use the hospital electronic system to record all proposals and interventions from ward rounding for each patient. These data allow us to research ward rounding in particular as other activities, such as medication reviews and medical error reports, were recorded separately.

### Study design

We designed a retrospective observational pre-post study that included all inpatients at the Ormoz Psychiatric Hospital between November 2019 and December 2020 for whom the clinical pharmacist provided recommendations. A control group or additional selection criteria were not used. The patients’ data were retrieved from electronic health records or hospital databases and were analyzed from the time the clinical pharmacist provided recommendations to three months after hospital discharge. Clinical outcomes were not measured.

Drug-related problems (DRPs) were classified according to the Slovenian classification of drug-related problems (DRP-SLO-V1) with some adjustments^12^. DRPs were identified as expressed (already observed) or potential (could occur in the future)^12^. Potential DRPs were described in terms of risk factors for them to arise in practice. We examined if any potential DRPs occurred by 3 months after discharge.

We identified problems related to treatment effectiveness, adverse events (treatment safety), and unnecessary drug treatment problems (e.g., no indication). The interventions were categorized into drug discontinuation, drug initiation, and drug regimen adjustment. The clinical pharmacist interventions were considered effective if risk factors for DRPs were not present at 3 months after discharge, based on the patients’ medical documentation. A similar methodology was used in our previous study in a nursing home setting^8^.

### Outcomes

The primary outcome measure was the acceptance rate (%) of interventions. The secondary outcome measures were the pharmacotherapy continuation rate 3 months after discharge (%) and the difference in the total number of DRPs by the time of hospital discharge. We only included clinical pharmacist interventions related to ward rounding.

### Data collection and analysis

The patient data were collected by Valentina Tement (MPharm student) and Matej Stuhec (first author), who has been working in a psychiatric hospital setting for over a decade. The baseline patient characteristics were calculated as the mean ± standard deviation as well as the difference before and after the interventions. SPSS Statistics 22.0 was used for statistical analysis. This study used the Strengthening the Reporting of Observational Studies in Epidemiology (STROBE) Statement for observational studies^13^. All methods were carried out in accordance with relevant guidelines and regulations.

### Ethical declaration

The study was approved by the National Medical Ethics Committee of the Republic of Slovenia in 2021 (Number 0120-18/2021/3; approved 11.3.2021). The Committee waived the requirement for informed consent, because it is a retrospective study.

## Results

### General results

The study included 224 patients (average age = 59.4, SD = 16.9, median = 56, 140 men and 104 women). The clinical pharmacist attended 75 ward roundings during the study period and gave 315 recommendations (4.2 per ward rounding). Nine recommendations with missing data were not included in the final analysis.

### Drug-related problems and outcomes

**Figure 1:**
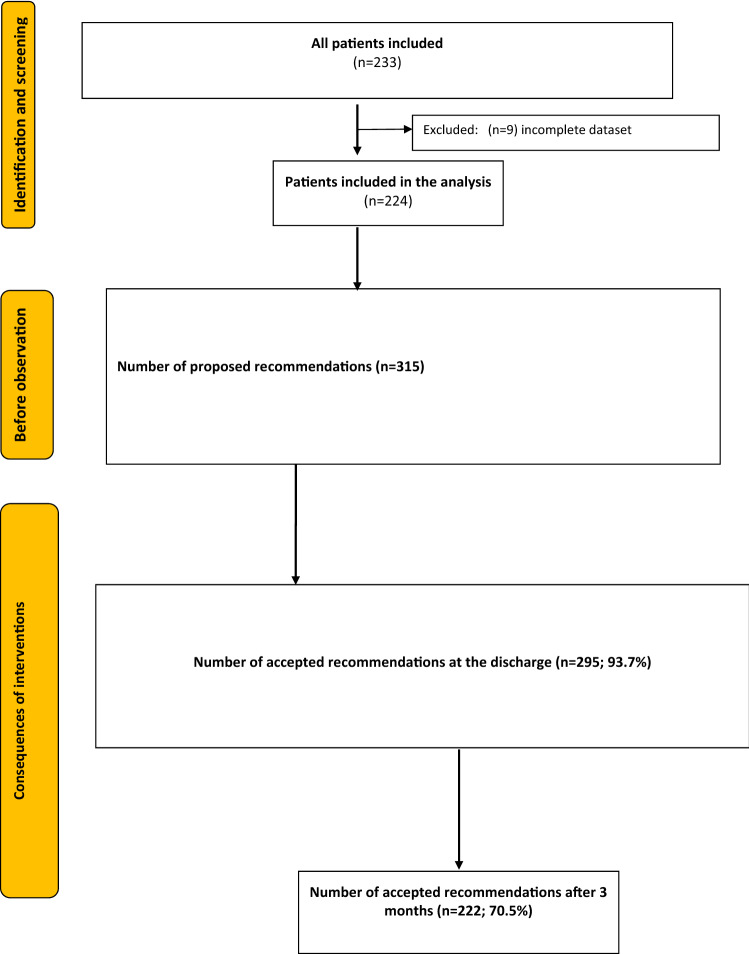
Flowchart of proposed and accepted interventions.

In total, the clinical pharmacist provided 315 separate recommendations, which were most commonly dose adjustments in 37.8% of all recommendations (*N* = 119), followed by medication initiation (24.4%, *N* = 77), medication discontinuation (24.4%, *N* = 77) and other interventions (13.3%, *N* = 42). The psychiatrists accepted 295 (93.7%) of them and rejected 20 (6.3%) proposed interventions (see Flowchart Fig. 1).

Out of the total 315 DRPs, 56.2% (*N* = 177) were expressed and 43.8% were potential (*N* = 138). After the clinical pharmacist recommendations, the number of both expressed and potential DRPs decreased by 93.8% to 11 and 9, respectively. See Table 1 for information on study outcomes.

**Table 1:** Study outcomes.

	Outcomes	Results
1	Acceptance rate (%)	93.7% (295/315 accepted interventions)
2	Drug-related problems (DRPs)	Clinical pharmacist most frequently provided recommendations at the psychogeriatric ward (40.6%, *N* = 128), followed by the addiction department (26.0%, *N* = 82), closed and open wards (22.6%, *N* = 71) and the daily hospital (9.8%, *N* = 31) Recommendations were most commonly given for antipsychotics (19.4%, N = 61) followed by antidepressants (16.8%, N = 53), antihypertensive medications (18.8%, N = 59), anxiolytics (8.2%, N = 26), antibiotics (7.0%, N = 22), hypnotics (4.1%, N = 13), and analgesics, antiepileptics and beta blockers (3.8% each, N = 12 each) and other medications (14.3%, N = 45) Out of the total 315 DRPs, 56.2% (N = 177) were expressed and 43.8% were potential DRPs (N = 138). Number of both expressed and potential DRPs decreased by 93.8% to 11 and 9 DRPs Treatment effectiveness (61.0% of all recommendations, *N* = 192, 91.2% acceptance rate), followed by inappropriate treatment (34.9%, *N* = 110, 97.2% acceptance rate) and medication safety (4.1%, *N* = 13, 100% acceptance rate)
3	Pharmacotherapy continuation rate 3 months after discharge (%)	Three months after discharge, 70.5% of all proposed recommendations were maintained, 28.6% were not continued and 1.0% had missing data

The recommended interventions were most commonly related to treatment effectiveness (61.0% of all recommendations, *N* = 192, 91.2% acceptance rate), followed by inappropriate treatments (34.9%, *N* = 110, 97.2% acceptance rate) and medication safety (4.1%, *N* = 13, 100% acceptance rate). No adverse events from the accepted recommendations were observed by the time of hospital discharge. The clinical pharmacist most frequently provided recommendations at the psychogeriatric ward (40.6%, *N* = 128), followed by the addiction department (26.0%, *N* = 82), closed and open wards (22.6%, *N* = 71) and the daily hospital (9.8%, *N* = 31).

Three months after discharge, 70.5% of all proposed recommendations were maintained, 28.6% were not continued and 1.0% had missing data. The DRPs were most frequently related to the treatment of mental disorders, followed by the treatment of cardiovascular diseases. In terms of medication type, recommendations were most commonly given for antipsychotics (19.4%, *N* = 61) followed by antidepressants (16.8%, *N* = 53), antihypertensive medications (18.8%, *N* = 59), anxiolytics (8.2%, *N* = 26), antibiotics (7.0%, *N* = 22), hypnotics (4.1%, *N* = 13), and analgesics, antiepileptics and beta blockers (3.8% each, *N* = 12 each) and other medications (14.3%, *N* = 45).

### Specific recommendations

#### Antibiotic therapy

Antibiotics were included in 22 different interventions (7.0%). See Table 2 for detailed information on treatment guidelines adherence and clinical pharmacist recommendations. All antibiotic treatments were effective and patients did not require additional treatment. The psychiatrists accepted all proposed recommendations.

**Table 2:** Proposed and accepted recommendations in antibiotic therapy; L(UTI): lower urinary tract infection; U(UTI): upper urinary tract infection.

Case number	Problem	Clinical pharmacists recommendations	Acceptance (yes/no)	Age
1a, 1b, 1c, 1d, 1e, 1f.	Ciprofloxacin treatment for L(UTI)	Cefuroxime was suggested	Yes	72 and 71 and 79 and 87 and 87 and 90
2	Levofloxacin treatment for L(UTI)	Cefuroxime was suggested	Yes	76
3	Antibiotic selection for L(UTI)	Amoxicillin treatment for L(UTI) in line with antibiogram results	Yes	64
4a, 4b	Antibiotic selection for L(UTI)	Nitrofurantoin was suggested	Yes	79 and 80
5	Fosfomycin for U(UTI)	Cefuroxime was suggested	Yes	79
6	Antibiotic selection for L(UTI)	Norfloxacin treatment for L(UTI) in line with antibiogram results	Yes	93
7 a, 7 b, 7c	Ciprofloxacin treatment for L(UTI)	Nitrofurantoin was suggested	Yes	67 and 68 and 68
8	Nitrofurantoin dose adjustment for L(UTI)	Dose adjustment	Yes	40
9	Co-trimoxazole treatment for L(UTI) together with quetiapine	Nitrofurantoin was suggested	Yes	49 and 50
10	Nitrofurantoin for L(UTI) and kidney failure	Cefuroxime was suggested	Yes	80
11	Moxifloxacin for chronic bronchitis	Amoxicillin was suggested	Yes	54
12 a, b, c	Other interventions (treatment duration, administration)	Different recommendations	Yes	49, 80, 90

#### Antipsychotic therapy

Antipsychotics were included in 61 different interventions (19.4%). See Table 3 for information on treatment guidelines adherence and clinical pharmacist recommendations.

**Table 3:** Proposed and accepted recommendations in antipsychotic therapy.

Case number	Problem	Age	Clinical pharmacists recommendations	Acceptance (yes/no)
1	Aripiprazole, levomepromazine and zuclopentixol	48	Aripiprazole discontinuation	Yes
2	Quetiapine and insomnia	41	Quetiapine dose adjustment	Yes
3	Sulpiride treatment	42	Sulpiride dose adjustment	Yes
4	Pregabalin treatment in patients with antipsychotics	66	Pregabalin dose adjustment	No
5a, 6b	Amisulpiride for acute psychotic episodes	45, 45	Amisulpiride initiation, Amisulpiride discontinuation	Yes, Yes
7a, 8b, 9c, 10d, 11e, 12f., 13g, 14h, 15i	Quetiapine treatment	62, 83, 35, 84, 39 , 38, 41, 40, 40	Quetiapine dose adjustment; Switching to risperidone; Quetiapine discontinuation; Quetiapine dose adjustment; Quetiapine discontinuation; Quetiapine SR initiation; Quetiapine dose adjustment; Quetiapine dose adjustment; Quetiapine dose adjustment	Yes, Yes, Yes, Yes, Yes, Yes, Yes, Yes, Yes
16a, 17b, 18c	Olanzapine treatment	45, 74, 59	Olanzapine dose adjustment; Switching to risperidone; Switching to quetiapine	Yes, Yes, Yes
19	Aripiprazole and sleep disorder	45	Monitoring and dose adjustment	Yes
20	Aripiprazole and olanzapine in patient with weight gain	45	Aripiprazole discontinuation and olanzapine initiation	Yes
21	Haloperidol and quetiapine	92	Haloperidol discontinuation	Yes
22	Quetiapine and dry mouth	64	Quetiapine discontinuation	No
23	Carbamazepine in the treatment of epilepsy	52	Dose adjustment	Yes
24	Quetiapine and sertraline treatment	54	Dose adjustment	Yes
25	No clear indication for Risperidone	80	Risperidone discontinuation	Yes
26	Sulpiride and in patient with QT prolongation	29	Combination discontinuation (monotherapy)	Yes
27	Risperidone and insomnia	78	Switching to quetiapine	Yes
28	Clozapine, olanzapine, aripiprazole	29	Olanzapine discontinuation	Yes
29	Clomethiazole and zolpidem in patient with dementia	80	Clomethiazole dose adjustment, zolpidem discontinuation, quetiapine initiation	Yes
30	Diazepam, quetiapine and clomethiazole	53	Diazepam discontinuation, quetiapine discontinuation	Yes
31	Quetiapine and blood count	84	Switching to zuclopentixol	Yes
32	Haloperidol and olanzapine and psychosis	30	Haloperidol discontinuation and switching to amisulpiride	Yes
33	Haloperidol, olanzapine and blood count	50	Olanzapine discontinuation	Yes
34	Aripiprazole and patient with psychosis	26	Switching to amisulpiride	Yes
35	Pregabalin and sulpiride concomitantly	49	Sulpiride discontinuation	No
36a, 37b	Sulpiride treatment	39, 45	Sulpiride dose adjustment	Yes, yes
38a, 39b	Risperidone and schizophrenia	81, 80	Risperidone dose adjustment; Risperidone dose adjustment	Yes, yes
40	Quetiapine and nitrazepam treatment	72	Monitoring and dose adjustment	Yes
41	Amisulpiride and patient with low blood pressure	60	Amisulpiride dose adjustment	Yes
42	Vortioxetine and sulpiride and depression	55	Switching vortioxetine to venlafaxine, quentiapine initiation, sulpirid discontinuation	Yes
43	Quentiapine in patient with QT prolongation	86	Switching to paliperidone	Yes
44	Risperidone, atomoxetine, quetiapine, diazepam, sodium valprote and patient with ADHD	38	Risperidone discontinuation, diazepam dose adjustment, sodium valproate dose adjustment	Yes
45	Quetiapine and hypersomnia	29	Quetiapine dose adjustment and monitoring	Yes
46	No clear indication for olanzapine	61	Olanzapine discontinuation	Yes
47	No clear indication for haloperidol	58	Haloperidol discontinuation	Yes
48	Patient with dementia and insomnia	85	Quetiapine initiation	Yes
49	Haloperidol and patient with heart attack	71	Switching to quetiapine	Yes
50–61	Other interventions which were not reported separately (e.g. monitoring)	Different age	Different interventions	Yes

*ADHD* attention deficit hyperactivity disorder, *SR* sustained release form.

## Discussion

This study is the first in Central Europe to examine clinical pharmacist interventions in interdisciplinary ward rounding at a psychiatric hospital. The results from Slovenia are widely applicable to countries with comparable healthcare systems, such as those in Central, Southeastern, and Eastern Europe. Our main finding is that the clinical pharmacist recommendations had a high acceptance rate and were maintained at follow-up.

The first important result is the high acceptance rate compared to medication review services. Pharmacist participation in interdisciplinary ward rounding was shown to be significantly associated with decreased medication errors/occupied bed/year in a study of over 1,000 US hospitals^10^. The 93.7% acceptance rate in this study is notably higher than in our two previous studies on a primary setting medication review service with an acceptance rate of 48.6% and 42.8%, in which suggestions were confirmed or rejected by general practitioners^14,15^. A higher acceptance rate of 88.0% was found in a 2014 study by Stuhec on a medication review service in a Slovenian psychiatric hospital^16^. While a medication review service alone may improve the quality of care received by psychogeriatric patients through reducing the number of medications, pDDIs, PIMs and improving treatment guideline adherence^14^, the differences in acceptance rates suggest that the benefits from clinical pharmacists may be increased if they are provided with greater access to psychiatrists, who are frequently the prescribers of psychotropics. This can be achieved either through closer collaboration in a hospital (as opposed to a primary care) setting or through direct inclusion in ward rounding. Most accepted recommendations were maintained 3 months after discharge, so the recommendations were positively accepted by psychiatrists and patients, although clinical outcomes were not measured.

Our second important finding is that pharmacist recommendations minimize DRPs. The pharmacist recommendations in this study addressed 56% of the expressed DRPs. The most frequent recommendations in our study were dose adjustments in 37.8% of cases (*N* = 119), followed by medication initiation 24.4% (*N* = 77), medication discontinuation 24.4% (*N* = 77) and other interventions (13.3%, *N* = 42). A 2019 study by Stuhec et al. had a similar distribution of intervention types and overall found that the clinical pharmacist identified several expressed DRPs, which had a positive impact on the patients’ quality of life, although the acceptance rate was lower (29.2%) than in our current study^8^. These differences may be due to how a clinical pharmacist is included in the treatment process in ward rounding as opposed to a medication review. During ward rounding, a clinical pharmacist can communicate with psychiatrists, patients, and nurses and can raise recommendations several times. The time from recommendations to action is longer in medication reviews, as the recommendations are sent out to the GP, who may choose to discuss them further with a psychiatrist. Furthermore, there was a difference in the interventions provided at different hospital departments. Most interventions (40.6%) were provided in the psychogeriatric ward, for patients treated with excessive polypharmacy. Studies in primary care settings similarly show that most clinical pharmacist recommendations were given for psychogeriatric patients^8,15^. Interventions at the addiction department constituted 26.0% of the interventions and were mostly benzodiazepine discontinuations or related to non-psychiatric diseases (e.g., hypertension).

The third important finding is that the clinical pharmacist in our study provided recommendations on pharmacotherapy in general and not exclusively on mental health disorders. While the latter were the most frequent, recommendations on cardiovascular, pain-related, and other conditions were also provided. This shows that a clinical pharmacist can be a versatile addition to the interdisciplinary medical team. All 22 antibiotic-related recommendations in our study were accepted. The clinical pharmacist recommended the discontinuation of quinolones (e.g., ciprofloxacin, levofloxacin, moxifloxacin) and cefuroxime and nitrofurantoin were added for lower urinary tract infection L(UTI). These recommendations are in line with treatment guidelines, because quinolones are discouraged as a first-line treatment, in particular for patients with mental disorders because of possible drug-drug interactions and adverse events^17,18^. Some combinations (e.g., with duloxetine) are even contraindicated with ciprofloxacin. Our findings on antibiotics are similar to a Slovenian prospective study in a psychiatric hospital, that found the most commonly prescribed antibiotics were co-amoxiclav (36.5% of cases) and co-trimoxazole (25.7%), ciprofloxacin (12.2%) and nitrofurantoin (6.80%), and detected important DDIs in 17 out of 74 patients (23% or 4.48/100 admissions), which were most frequently the combination of co-trimoxazole and quetiapine, and of ciprofloxacin and olanzapine^17,19^. The antibiotic-related pharmacist recommendations in our study were evidence-based, as the first-line treatment for psychogeriatric patients was cefuroxime (few DDIs and few adverse events) and nitrofurantoin (efficacy, few DDIs). The safest antibiotics for patients with mental disorders are penicillins, cephalosporins, and nitrofurantoin; macrolides and quinolones should be used with great caution^18,19^. The recommendations were also effective insofar as infections did not reoccur 3 months after discharge.

The fourth important finding is that the recommendations in our study were most frequently related to antipsychotics. The acceptance rate was much higher than in previous studies in primary care settings, which may be due to a different working environment^13,14^. Interestingly, many pharmacist interventions were connected with antipsychotic polypharmacy (APP) use, which is not recommended in the treatment guidelines^20,21^. Our study found APP combinations of aripiprazole, levomepromazine and zuclopenthixol, of olanzapine with aripiprazole or haloperidol, and of quetiapine with haloperidol, sulpiride, clozapine or risperidone. The clinical pharmacist recommendations were evidence-based as APP should only be considered after clozapine treatment when possible^18–23^. The results show that the clinical pharmacist reduced the use of APP and often suggested antipsychotic discontinuation in cases of excessive APP, in line with the results by Suzuki et al. that show APP may be replaced with antipsychotic monotherapy in most cases^22^. A Finnish nationwide cohort study of schizophrenia patients found that the hazard ratio of psychiatric rehospitalization was 7% lower during any polypharmacy than any monotherapy period (HR, 0.93; 95% CI, 0.91–0.95; *p* < 0.001), but that the difference may be clinically insignificant. Aripiprazole with clozapine was associated with the lowest risk of rehospitalization, so polypharmacy may be a feasible treatment option for schizophrenia (e.g., clozapine with aripiprazole, any long-acting antipsychotic with olanzapine, and clozapine with olanzapine). However, the results for clozapine monotherapy were not substantially worse than those of the best polypharmacy option^25^. Many interventions were focused on the indication for antipsychotics in insomnia treatment and were in line with the clinical guidelines, according to which quetiapine and olanzapine are not first-line treatments because of weak evidence and possible adverse events^26,27^. Adverse events from antipsychotics are frequent^26,28 ^ and even more important in elderly patients, who tend to have multiple diseases. The results of our study show that the clinical pharmacist often suggested olanzapine discontinuation in APP (or in cases without indication), which can reduce the risk of important cardiovascular adverse events in long-term treatments (e.g., weight gain, QT prolongation and diabetes). These interventions were also maintained 3 months after discharge, suggesting they were effective and well tolerated.

This study also has several limitations, mostly due to the selection criteria, minimum exclusion criteria and non-randomized conditions. The study is retrospective and non-randomized, so causal relationships cannot be established. Patients were not monitored over a longer period of time (e.g., eight months), limiting the scope of our results. Another limitation is the population heterogeneity that increases intervariability as we included patients with various mental disorders. Another source of selection bias stems from the inclusion of patients with polypharmacy and comorbidities. The sample size used in this study was small and not pre-calculated. We also did not measure clinical outcomes and interventions other outside of ward rounding (e.g., medication review). These limitations could be addressed with prospective studies in real clinical settings. To the best of our knowledge, this is the first study in Central Europe to examine the impact of clinical pharmacists in interdisciplinary team rounding at a psychiatric hospital.

## Conclusions

Clinical pharmacist participation in interdisciplinary team rounds at a psychiatric hospital led to fewer drug-related problems with a very high acceptance rate. Their recommendations were mostly evidence-based and maintained at a 3-month follow-up. More European countries across could adopt this practice to improve outcomes for patients with mental disorders in psychiatric hospitals. Additional research with larger samples or a prospective design would be needed to replicate the positive results.
